# Process, Rationale, and Interventions of Pakistan’s National Action Plan on Chronic Diseases

**Published:** 2005-12-15

**Authors:** Sania Nishtar, Khalif Mohamud Bile, Ashfaq Ahmed, Azhar M.A. Faruqui, Zafar Mirza, Samad Shera, Abdul Ghaffar, Fareed A Minhas, Aslam Khan, Naeem A Jaffery, Majid Rajput, Yasir A Mirza, Mohammad Aslam, Ejaz Rahim

**Affiliations:** Heartfile; World Health Organization (WHO), Islamabad, Pakistan; Office of International Health, Ministry of Health, Islamabad, Pakistan; National Institute of Cardiovascular Diseases, Karachi, Pakistan; Network for Consumer Protection, Islamabad, Pakistan; Diabetes Association of Karachi, Karachi, Pakistan; Global Forum for Health Research, Geneva, Switzerland; WHO Collaborating Centre for Psychiatry, Rawalpindi, Pakistan; Military Hospital, Rawalpindi, Pakistan; Ziauddin Medical University, Karachi, Pakistan; Office of the Director General Health, Ministry of Health, Islamabad, Pakistann; Communications Department, Heartfile, Islamabad, Pakistan; Communications Department, Heartfile, Islamabad, Pakistan; Office of the Secretary of Health, Ministry of Health, Islamabad, Pakistan

## Abstract

Most developing countries do not comprehensively address chronic diseases as part of their health agendas because of lack of resources, limited capacity within the health system, and the threat that the institution of national-level programs will weaken local health systems and compete with other health issues. An integrated partnership-based approach, however, could obviate some of these obstacles.

In Pakistan, a tripartite public–private partnership was developed among the Ministry of Health, the nongovernmental organization (NGO) Heartfile, and World Health Organization. This was the first time an NGO participated in a national health program; NGOs typically assume a contractual role. The partnership developed a national integrated plan for health promotion and the prevention and control of noncommunicable diseases (NCDs), which as of January 2006 is in the first stage of implementation. This plan, called the National Action Plan on NCD Prevention, Control, and Health Promotion (NAP-NCD), was released on May 12, 2004, and attempts to obviate the challenges associated with addressing chronic diseases in countries with limited resources. By developing an integrated approach to chronic diseases at several levels, capitalizing on the strengths of partnerships, building on existing efforts, and focusing primary health care on chronic disease prevention, the NAP-NCD aims to mitigate the effects of national-level programs on local resources.

The impact of the NAP-NCD on population outcomes can only be assessed over time. However, this article details the plan's process, its perceived merits, and its limitations in addition to discussing challenges with its implementation, highlighting the value of such partnerships in facilitating the missions and mandates of participating agencies, and suggesting options for generalizability.

## Background

Chronic noncommunicable diseases (NCDs) are estimated to have caused 33.4 million deaths worldwide in 2002; of these, 72% occurred in developing countries (1). In Pakistan, chronic diseases (cardiovascular disease, diabetes, chronic lung diseases, and cancer) are among the top 10 causes of morbidity and mortality and account for approximately 25% of total deaths (2,3). Thirty-three percent of the adult population older than 45 years has high blood pressure; 10% of the adult population older than 18 years has diabetes, and more than 54% of men use tobacco. Data from an unselected autopsy series have shown coronary artery involvement (of greater than 50% luminal diameter reduction) in more than 24% of those studied. Moreover, the coastal metropolis of Karachi, with a population of more than 15 million, reports one of the highest incidences of breast cancer for any Asian population (4-9). Over the years, Pakistan's federal and provincial Ministries of Health have been heavily burdened with reproductive-health and infectious-disease issues. However, Pakistan is undergoing an epidemiological transition and, hence, is now also focusing on chronic diseases.

Pakistan has a population of 150 million and an annual gross national product (GNP) per capita of U.S. $700; during the last 10 years, 0.6% to 0.8% of its GNP and 5.1% to 11.6% of its development budget has been spent on the health sector (2). Seventy percent of clinical services are delivered by private-sector health care providers, and out-of-pocket payments are the major source of health financing, despite the existence of an extensive public-sector–owned health care system. Preventive and educational services are delivered almost exclusively by the public sector. Lately, as part of certain preventive programs (HIV and AIDS programs in particular), nongovernmental organizations (NGOs) have been delivering preventive care, albeit in a contractual role in which NGOs enter into contracts with the public sector.

As in most other developing countries, NCDs had not featured prominently on Pakistan's health agenda until 2003, when a national integrated plan for health promotion and the prevention and control of NCDs, known as the National Action Plan on NCD Prevention, Control, and Health Promotion (NAP-NCD), was initiated. NAP-NCD attempts to obviate the challenges associated with addressing chronic diseases in countries with limited resources. Initially, an agreement was developed between the Ministry of Health and Heartfile, an Islamabad, Pakistan-based nonprofit, NGO focused on chronic disease prevention and control and health promotion; a month later, the World Health Organization (WHO) was asked to join the initiative. Heartfile's role in the NAP-NCD was to advocate for increased focus on chronic disease in the national health agenda. This was the first time an NGO had participated in a national health program in more than a contractual role.

The partnership, developed on a national level, was mandated with the task of developing and implementing a strategic plan to prevent and control the rates of NCDs (10). The NAP-NCP was released on May 12, 2004, within a year of the agreement's signing, and as of January 2006 is in its first phase of implementation (11,12).

## Developing the Plan

Chronic diseases generally are linked by common risk factors and include cardiovascular disease (CVD), diabetes, cancer, and chronic lung disease. However, the NAP-NCD also includes injuries and mental illness in its framework because of government requirements.

A three-stage process was used to develop the NAP-NCD: 1) planning within the disease categories, 2) setting priorities, and 3) developing an integrated approach to preventing NCDs (13).

Next, a situational analysis was conducted in which data on current epidemiological evidence for NCDs were gathered, existing strategies and policy measures were summarized, gaps in the system and opportunities that existed for integration with existing programs were outlined, and the potential for program implementation was analyzed. Then, a broad-based consultative process was established, which included health professionals, NGOs, professional societies, community representatives, donor and development agencies, corporations, and legislators, and priority action areas were identified. In the absence of local cost-effectiveness data, other priority-setting criteria were used, such as the extent to which an intervention was locally feasible, promoted community empowerment and participation, built on the strengths of partnerships, and contributed to capacity building and health systems strengthening. In addition, the capacity of the public health system and the ability of health care leaders to implement the NAP-NCD were also identified as important criteria.

Finally, a tool called the Integrated Framework for Action (IFA) was developed to identify action items that could be applied to all NCDs  (14). (The IFA is available from http://heartfile.org/pdf/IFAPDF.htm.) Additionally, the IFA included two sets of strategies — those that were common to all NCDs (Table 1) and those specific to each NCD (Table 2). The first set of strategies includes behavioral-change communication, focusing health services on NCDs, development of institutional mechanisms, and monitoring and surveillance; the second set covers legislative or regulatory matters and research. The IFA — which also provides guidance to administrators and health policy planners — helps set national goals at process, output, and outcome levels; defines integrated actions to meet those goals; and allows for program assessment.

## Components and Configuration of the NAP-NCD

The NAP-NCD prioritizes a population-based approach to chronic diseases that encompasses public education, behavioral-change communication, legislation, and regulation. These approaches have the greatest potential to reduce NCD risk and uphold the principles of WHO's "Health-for-All Policy for the 21st Century" because the high-risk approach (i.e., targeting individuals at high risk for chronic disease rather than populations as a whole) may be inaccessible to the majority of the country's underprivileged population. Thirty-two percent of Pakistan's population is below the poverty level of U.S. $1 a day (15). The NAP-NCD will be implemented in two phases.

### First phase

The first phase of implementation, which spans 3 years (May 2004 through July 2006), is jointly funded by the Ministry of Health, Heartfile, and WHO. The implementation status is reviewed for accountability and program evaluation every 3 months, and progress is posted online (24); the process and output indicators stipulated in the IFA are used for process evaluation. The first phase of the NAP-NCD's implementation focuses on the action items summarized in Tables 1 and 2 and is organized into the following three priority areas: an integrated and sustainable population-based NCD surveillance system, an integrated behavioral-change communication strategy, and legislation in key areas.

An integrated and sustainable population-based NCD surveillance system is a prerequisite for effective planning, implementation, and evaluation of NCD prevention programs (with the exception of cancer, because a registry has to be used for its surveillance) and is regarded as an entry point for activities related to the prevention of NCDs — an approach validated in several settings (16,17). The NAP-NCD's surveillance model includes population surveillance of primary NCD risk factors (poor diet, physical inactivity, and smoking) and combines modules on population surveillance of injuries, mental health, and stroke. In addition, the model has been adapted for program evaluation, which enables it to use indicators to track implementation processes and facilitates an assessment of how interventions work and which components are the most successful. An initial cross-sectional survey with a sample of sufficient size and the power to detect population-level changes over time of the risk factors and NCDs has been conducted (18,19).

The integrated behavioral-change communication strategy consisted of two interventions. The first intervention included a media campaign targeting 90% of the country's population. The second intervention introduced chronic disease prevention into the work plan of Lady Health Workers (LHWs) — Pakistan's field force of more than 83,000 grassroots health caregivers.

For the media campaign, 30-second spots and 5-minute programs are being aired for 2 years during prime time on national television and radio and began in May and June 2005. One announcement focuses on creating awareness about high blood pressure by advocating opportunistic screening (i.e., using every clinical encounter "opportunity" to check the blood pressure of every patient); the other emphasizes the principles of cardiovascular disease prevention.

Until recently, LHWs were involved in delivering reproductive-health– and communicable-disease–related services to poor and underprivileged households in rural areas covering 50% of Pakistan's population. Heartfile had previously pilot tested an approach in which CVD prevention was introduced into its work plan in the Lodhran district as part of a CVD prevention demonstration project. Seven hundred LHWs were involved in this pilot project from 2001 through 2003 (20,21). Lessons learned from this experience have enabled the introduction of the chronic disease perspective into the training module in addition to an increased focus on chronic disease in 17 other districts as part of the NAP-NCD.

Other priority areas for the first phase of implementation include lobbying for key legislative actions, identifying research areas, building capacity within the health system, and focusing on institutional measures.

### Second phase

The second phase of implementation will broaden the scope to include measures that focus health services on prevention; the launch of the second phase is planned for 2006. The second phase will have implications for training and capacity building of health professionals, improving basic infrastructure, and ensuring availability and access to certain drugs at all levels of health care.

Health care delivery in Pakistan is characterized by a variety of roles played by different categories of health care providers, and all will be drawn into the loop. The NAP-NCD makes recommendations to ensure physician training as a permanent function of the health care system by establishing links with provincial and district health departments; it also makes recommendations on how to form a comprehensive continuing medical education program structured around broad-based prevention-related goals and objectives to ensure ongoing training for both private-sector and pubic-sector physicians. The second phase of the NAP-NCD will also include other legislative actions.

## Merits and Limitations of the Approach

The NAP-NCD presents one approach to developing a national strategy for chronic diseases in countries with limited resources. The strategy includes integration at six distinct levels. By *grouping chronic diseases* and *integrating actions*, there is a shift from a national-level approach to an approach based on diseases, which has significant implications for maximizing health care resources. Horizontally integrating actions with existing initiatives strengthens the public health system; adopting integrated models on surveillance and behavioral-change communication in addition to focusing health services on NCDs will yield important empirical evidence for emerging chronic disease programs. Additionally, integrating health promotion and prevention within the same program achieves two objectives for two populations with common activities. 

The evaluation mechanism of this model, which is structured within the accountability and progress charts of the IFA, allows program assessment at a process-and-outcomes level and assessment of the level of contribution partners have made to achieve the NAP-NCD's objectives.

This program is of value to all partners. By leveraging the strengths of the nonprofit private-sector technical partner (Heartfile), the government included NCD prevention in its policies. NGOs and the civil society can contribute to achieving national goals; however, this potential remains largely untapped. This model provides a mechanism for engaging NGOs in the national decision-making process and ensures their participation both in the formulation of health policy and implementation of national plans. Though an evaluation mechanism, it also enables the assessment of each partner's contribution to achieving objectives. In this model, WHO is gaining experience with a model in which WHO resources — which are otherwise allocated for the public sector — support the private sector in a national model. The partnership is therefore integrated with national health priorities and complements state initiatives.

This program is one of the few examples of a public–private partnership for chronic disease prevention, an area that has largely remained unexplored as part of global efforts to build public–private partnerships. This program's implementation is expected to yield important information about the performance of the health system by building chronic disease partnerships in evidence-based models. As for infectious disease partnerships, ethical, methodological, accountability, sustainability, and governance issues must be considered (22-25).

This initiative also created a mechanism for visible involvement and participation of many other stakeholders in the national consultation process in addition to avenues for their participation in the process of implementation. This is important because many factors that affect NCDs are outside of the health sector domain; these include trade, agriculture, finance, education, and communication. However, there is also the need to fit this strategy within a more explicit policy framework — one that makes it obligatory to link relevant health ministries in a manner that is mutually supportive of national NCD goals. The program needs to be supported by a clear, strong, and sustained political commitment.

The NAP-NCD can serve as both an empirical basis for an integrated approach to NCDs and an experimental basis of health sector reform in the area of public–private collaboration; most developing countries have limited experience with each. It is also likely to yield useful lessons for ministries of health, NGOs, and multilateral agencies for establishing chronic disease programs in developing countries.

## Figures and Tables

**Table 1:** Action Items for Prevention of All Noncommunicable Diseases (NCDs) in the National Action Plan on NCD Prevention, Control, and Health Promotion (NAP-NCD), Pakistan

**Action items**
Development and maintenance of an integrated population-based NCD surveillance system incorporating program monitoring and evaluation components^a^
Development of a research-guided, behavioral-change communication strategy for NCDs; implementation at the national level through the media and at the grassroots level through community health workers (Lady Health Workers)^a^
Development and implementation of a sustainable, scientifically valid, culturally appropriate and resource-sensitive continuing medical education program for professional education and involvement of all categories of health care providers in NCD prevention and integration in health services
Upgrading infrastructure in health care facilities and ensuring availability of essential drugs relevant to chronic disease prevention at the basic health care level
Building capacity of health systems in support of cardiovascular disease (CVD) prevention and control
Building a coalition or network of organizations at the national, provincial, and local levels facilitated by federal and provincial health services to add momentum to CVD prevention and control as part of a comprehensive NCD prevention effort

a Priority actions.

**Table 2:** Action Items to Address Individual Noncommunicable Diseases (NCDs) in the National Action Plan on NCD Prevention, Control, and Health Promotion (NAP-NCD), Pakistan

**Priority Areas**	**Action Items**
**Policies and legislation**	Effectively implement existing legislation on mental health — expanding its base to protect the interest of special groups such as prisoners, refugees and displaced individuals, women, children, and individuals with disabilities
	Revisit policy on diet and nutrition to expand its focus on undernutrition; establish policies and strategies to limit the production of, and access to, *ghee* as a medium for cooking and agriculture; and establish fiscal policies that increase the demand for healthy foods and make healthy food more accessible
	Develop a physical activity policy
	Institute legislation for occupational health and safety
	Enforce seatbelt and helmet laws effectively
	Upgrade existing highway ordinances
	Enact and enforce legislation for locally manufactured vehicles
	Regulate drivers' training and licensing
	Enforce legislation on building safety
	Strive to improve trauma care to the extent that a credible, cost-effective analysis suggests
	Enforce the National Environmental Quality Standards strictly and transparently
	Institute proactive measures to contain potential risks to cancer in industrial settings; enforce labor laws more strictly
	Regulate chemical handling stringently
	Incorporate preventive health in the mandate of organizations providing health coverage for the labor workforce in order to contain exposure to carcinogenic agents in the environment and in worksites
	Develop a price policy for tobacco products
	Subject tobacco to stringent regulations governing pharmaceutical products
	Initiate fiscal measures to reduce dependence on revenues generated from tobacco
	Initiate measures for agricultural diversification with respect to tobacco cultivation and assisting with crop diversification
	Enforce legislation on smuggling tobacco, contrabands, and counterfeiting
	Enforce more strict legislation to phase out all types of tobacco advertising
	Regulate the import of areca nut
**Research**	Identify causal associations specific to the population in NCDs to define precise targets for preventive interventions
	Use clinical endpoint trails to define the best therapeutic strategies for prevention of NCDs, weighing cost against economic feasibility
	Research local policy and operations to examine tobacco tax policies in addition to marketing and advertising strategies
	Use existing data sources to assess cancer trends in industrial settings that may be exposing people to carcinogenic agents
	Conduct studies to bridge critical gaps in evidence of appropriate and cost-effective strategies for preventing common cancers in Pakistan
	Identify black spots on highways and on city roads; assessments guide interventions appropriate to reduce the risk of highway crashes in these settings
	Evaluate interventions to reduce all forms of violence in Pakistan
	Examine trends in outdoor air pollution levels and their determinants to develop appropriate public health interventions; address other priority areas such as conducting research to quantify the magnitude and determinants of chronic lung diseases attributable to indoor air pollution both in rural and urban areas and developing appropriate public health strategies to reduce risks in such settings
**Institutional mechanisms**	Establish an Occupational Safety and Health Association
	Establish a road safety committee
	Establish a National Safety Commission
	Develop product safety standards
	Establish standards for household useables
	Establish a National Cancer Control Council
	Sustain institutional support of established cancer registries to facilitate surveillance
